# Loss of Olfactory Receptor Function in Hominin Evolution

**DOI:** 10.1371/journal.pone.0084714

**Published:** 2014-01-02

**Authors:** Graham M. Hughes, Emma C. Teeling, Desmond G. Higgins

**Affiliations:** 1 UCD School of Biology and Environmental Science, University College Dublin, Belfield, Dublin, Ireland; 2 UCD Conway Institute of Biomolecular and Biomedical Research, University College Dublin, Belfield, Dublin, Ireland; University of Illinois at Urbana-Champaign, United States of America

## Abstract

The mammalian sense of smell is governed by the largest gene family, which encodes the olfactory receptors (ORs). The gain and loss of OR genes is typically correlated with adaptations to various ecological niches. Modern humans have 853 OR genes but 55% of these have lost their function. Here we show evidence of additional OR loss of function in the Neanderthal and Denisovan hominin genomes using comparative genomic methodologies. Ten Neanderthal and 8 Denisovan ORs show evidence of loss of function that differ from the reference modern human OR genome. Some of these losses are also present in a subset of modern humans, while some are unique to each lineage. Morphological changes in the cranium of Neanderthals suggest different sensory arrangements to that of modern humans. We identify differences in functional olfactory receptor genes among modern humans, Neanderthals and Denisovans, suggesting varied loss of function across all three taxa and we highlight the utility of using genomic information to elucidate the sensory niches of extinct species.

## Introduction

Neanderthals, whose fossils have been found throughout Eurasia, are extinct relatives of modern humans. Neanderthals disappeared from the fossil record approximately 30,000 years ago and a number of hypotheses exist to explain why [1]. An analysis of the 3D structure of the Neanderthal cranium shows a significant size difference between the olfactory bulb of modern humans and Neanderthals, with human olfactory bulbs being larger, suggesting a potential difference in olfactory capabilites between Neanderthals and modern humans [2]. However, a recent study concluded that although there are differences in the size of the olfactory bulb among living humans (smokers versus non-smokers), there was little to no difference detected in the ability to differentiate odors, suggesting that odor detection thresholds may be more important than bulb size [3]. Therefore, although the difference in bulb volume between modern humans and Neanderthals may represent varying olfaction capabilities in the two lineages, morphology alone may not be enough to determine this. Isotope analysis of Neanderthal fossilised teeth suggests a diet high in protein with some plant consumption [4]. Fossils of another hominin, the extinct Denisovan hominin - a sister taxon to Neanderthals, have been found in the Denisova cave in Siberia [5]. Almost no phenotypic information exists for this species, however both Neanderthal and Denisovan genomes have recently been sequenced allowing further exploration of their genomes [1], [6].

Olfactory receptors (ORs) are G protein-coupled receptors located in the olfactory epithelium of the nasal passage, encoded by the largest mammalian gene family [7]. ORs are a highly plastic gene family showing frequent gene birth/loss events [7]. Two OR classes have been described, Class I and II which bind water and air borne odours respectively [8]. ORs are grouped into subfamilies based on sequence similarity [8]. A ‘many – to – many’ binding relationship exists between odorant molecules and ORs, with a single odorant molecule binding to many receptors and a single receptor binding many odorant molecules [21]. The 1000 Genomes project revealed that SNPs can lead to differences in functional and non-functional ORs between modern humans, including instances of ‘rescue’ from pseudogene to functional gene [9]. Loss and gain of OR function correlates with ecological niche adaptation as observed by the high numbers of pseudogenes in cetaceans, assumed driven by adaptation to aquatic environments [8].

Given the physical changes observed between the Neanderthal and modern human olfactory bulb [2] we examined whether OR gene losses vary across modern humans, Neanderthals and Denisovans representing differences in the evolution of olfaction in hominin species.

## Materials and Methods

### Olfactory Receptor Data

853 annotated modern human ORs were downloaded from the HORDE (Build #43) online database [10]. Of these, 466 are annotated pseudogenes. 813 chimpanzee ORs, 433 of which are pseudogenes, have been previously annotated by Go and Niimura [11]. We compared non-functional ORs between the two to identify conserved stop codons present in both species. A stop codon was considered conserved if it was present in the same position in modern human and chimpanzee. Chimpanzee pseudogenes were assigned to one of the 13 OR subfamily clusters, OR 1/3/7, OR 2/13, OR 4, OR5/8/9, OR 6, OR 10, OR 11, OR 12, OR 14, OR 51, OR 52, OR 55 and OR 56, using methods described in Hayden et al. 2010 [8]. OR pseudogenes shared between the chimpanzee and modern human genomes were also assumed to be non-functional in the Neanderthal and Denisovan genomes despite limited or no read coverage [11].

### Hominin Genome Alignments

Alignments of the 1× Neanderthal genome, the recently sequenced 50× Altai Neanderthal (http://www.eva.mpg.de/neandertal/index.html), and both the 1× and 30× Denisovan genomes with the modern human genome were initially in BAM format and were subsequently converted to SAM format via SAMtools [1], [12]. Neanderthal reads were parsed and mapped to the database of modern human ORs using Bowtie [13]. Allowing a difference of n = 3 nucleotides per read, a total of 59,695 and 3,737,906 reads were mapped for the 1× and 50× Neanderthal genomes respectively. Denisovan reads were also mapped to the modern human OR dataset with Bowtie, allowing n = 3 mismatches. For the Denisovan genomes, 84,867 reads (1×) and 8,110,291 reads (30×) were mapped to the reference modern human ORs. A gene was considered non-functional if it contained an ‘in-frame’ stop codon [14].

**Figure 1:**
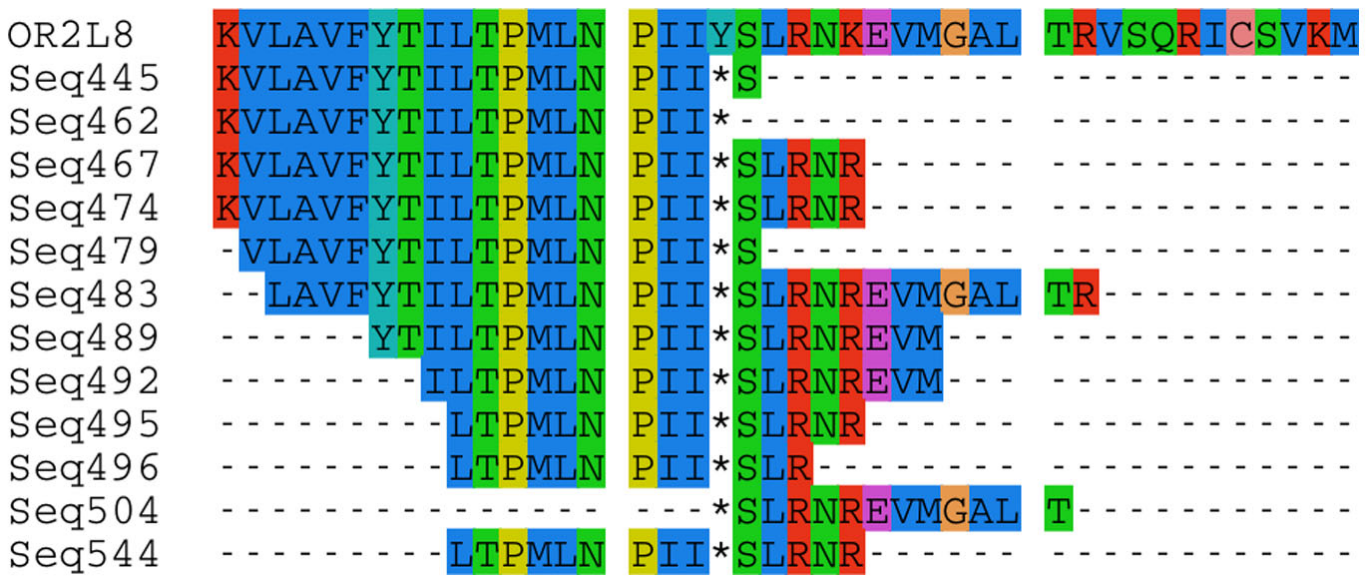
Reads from extinct hominin species have stop codons at the same position in at least 2 reads and are therefore designated as ‘non-functional’. Extinct hominin reads were mapped to all genes in the human OR reference database (HORDE). Here, they are mapped against the modern human OR2L8 functional sequence in the top line. Unique sequence identifiers were used to avoid potential multi-locus read mapping. Some mutations have been found shared across all three hominins while some are unique losses.

Putative Neanderthal and Denisovan OR reads were aligned to the modern human OR gene family using Clustal Omega [15]. These reads were translated into amino acids using the open reading frame with respect to the target modern human OR. Due to the possibility of sequencing error leading to stop codons in the ancient genomic data, a stop codon needed to be present in at least 2 reads mapping to the same position to be considered a potential indicator of OR loss of function. This is depicted in Figure 1. Alignments between modern human and extinct hominins were grouped into the following 4 categories: (1) A stop codon present in at least 2 or more ancient hominin reads, that mapped to the same position in a functional modern human OR (functional in modern human lineages); (2) A stop codon present in at least 2 reads that maps to the same position in a non-functional modern human OR (non-functional in both); (3) Stop codons present in reference modern human OR not found in ancient hominin reads mapping to the same locus (non-functional in the modern human lineage); (4) functional in both taxa.

To further confirm mutations that lead to the gain or loss of function in the Neanderthal and Denisovan, both the modern human reference OR and hominin reads were realigned using the Genome Analysis ToolKit (GATK) worfklow and variant calling tools [16], [17]. For each potential mutation identified using the above methodology, GATK also identified said loss of function. To account for potential deamination in the ancient DNA samples that could result in a false functionality assumption we compared the ORs in the high coverage genomes to the low coverage genomes, looking for the same mutation at the same position indicating that the mutation was real.

### Multi-locus Read Mapping

Due to the high degree of conserved sequence similarity between ORs in the same subfamilies, there was a possibility of reads mapping to multiple loci for both the Denisovan and Neanderthal genomes, inflating the number of genes unique to each lineage. To overcome this issue when identifying non-sense mutations, we only used reads that shared a distinct unique sequence with a single target OR. In cases where an amino acid sequence was not phylogenetically divergent enough due to redundancy in the genetic code, a nucleotide sequence was used. The sequences used for each resulting OR of interest are described in Table 1.

**Table 1:** Comparison of extinct hominin ORs with their modern human homolog.

Species	Receptor	Mutation		Mutations in modern humans?	Unique identifier
		Nucleotide	Amino acid		
**Neanderthal non-functional**	OR2L8	T->A	Y->*	Identical mutation is found in certain humans	ILTPMLNPII.SLRNRE
	OR4X1	T->A	Y->*	Identical mutation is found in certain humans	IVAVF.TVVT
	OR5AR1	C->T	Q->*	Identical mutation is found in certain humans	.DPQME
	OR5M11	T->A	Y->*	Identical mutation is found in certain humans	RYVAI.DPLRYS
	OR5P3	C->G	Y->*	Unique loss	KSS.STD
	OR6C74	C->T	R->*	Identical mutation is found in certain humans	LHLKTPMYFFL.NFSFL
	OR7A5	C->G	Y->*	Unique loss	AILGV.LSS
	OR8I2	C->G	Y->*	Identical mutation is found in certain humans	TIVIPMLNPLI.SLRNKD
	OR10X1	G->A	W->*	Identical mutation is found in certain humans	GLT.VDRS
	OR13C4	T->G	Y->*	Different loci, known variant in humans	FFM.AKPKSQDLL
**Neanderthal functional**	OR1P1P	T->A	*->K	Identical mutation is found in certain humans	S.CS.ILP
	OR2J1P	T->C	*->Q	Identical mutation is found in certain humans	GTTGATACC.AGGCA
	OR9H1P	T->C	*->Q	Identical mutation is found in certain humans	MQGR.KAF
**Denisovan non-functional**	OR2L8	T->A	Y->*	Identical mutation is found in certain humans	ILTPMLNPII.SLRN
	OR2S2	C->G	Y->*	Different loci, known variant in humans	LILLM.LVILL
	OR2T8	T->G	Y->*	Different loci, known variant in humans	SFP.CGAHEIDHFFCETP
	OR4C12	G->A	W->*	Unique loss	IRKL.RKKVT
	OR5AC2	C->T	R->*	Different loci, known variant in humans	VSLLL.LTFFR
	OR5M11	T->A	Y->*	Identical mutation is found in certain humans	I.DPLRYS
	OR8I2	C->G	Y->*	Identical mutation is found in certain humans	IVIPMLNPLI.SLRNKDVK
	OR51Q1	C->T	R->*	Identical mutation is found in certain humans	AE.LRA
**Denisovan functional**	OR1P1P	T->A	*->K	Identical mutation is found in certain humans	S.CS.ILP
	OR2J1P	T->C	*->Q	Identical mutation is found in certain humans	GTTGATACC.AGGCA
	OR5E1P	A->C	*->C	Unique gain	FATTE.FLL
	OR9H1P	T->C	*->Q	Identical mutation is found in certain humans	MQGR.KAF
	OR13C1P	T->C	*->R	Unique gain	L.YPIIMSKA

## Results and Discussion

**Figure 2:**
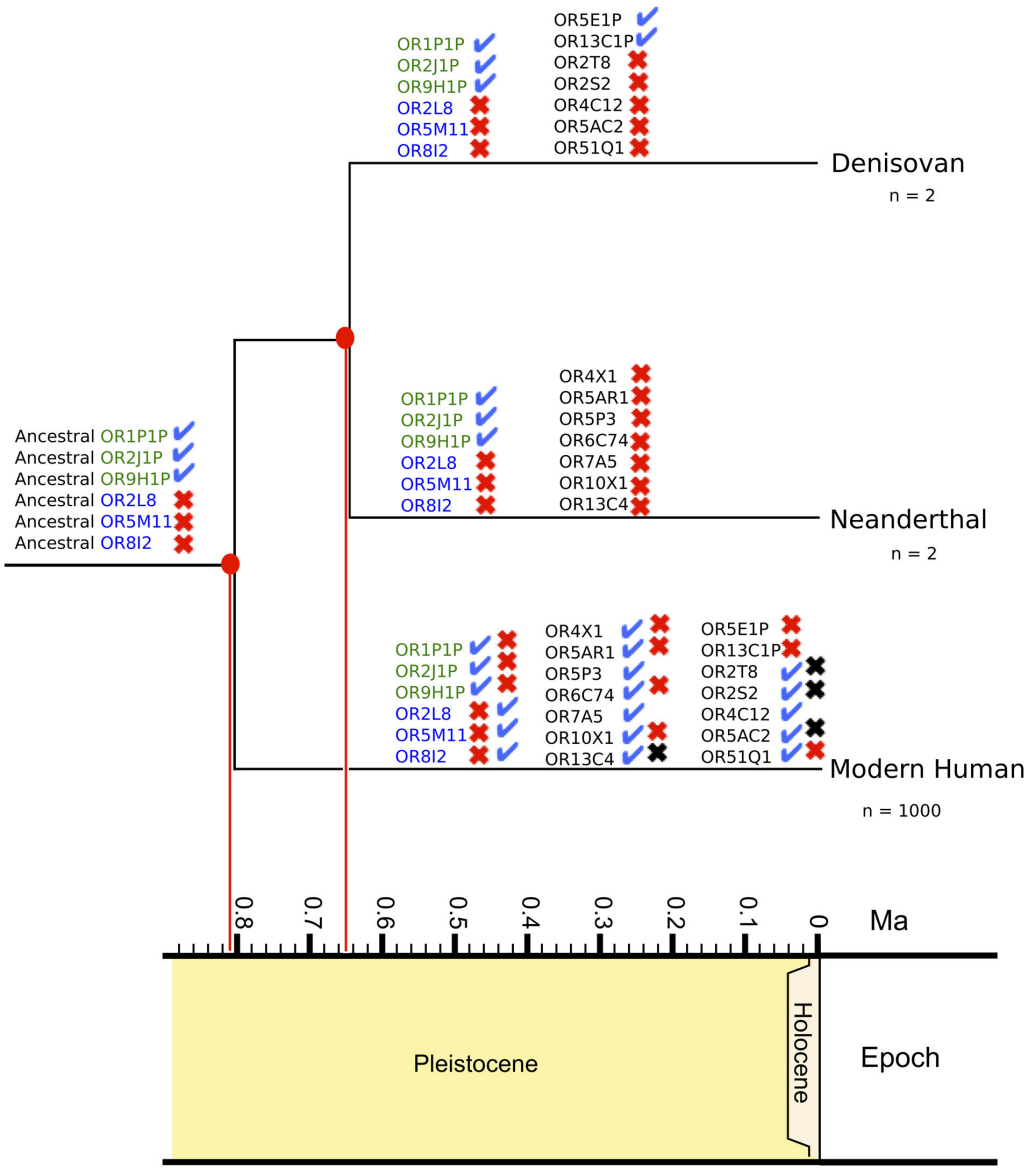
The phylogenetic relationships and divergence times [5] (indicated by red lines) across modern human, Neanderthal and Denisovan are highlighted. Varying functional and non-functional ORs between each species are shown along each lineage. Losses at identical positions (red ‘X’) and different positions (black ‘X’) are displayed. ORs for which the ancestral state is inferred are displayed at the root of the tree, with ancestral functional and non-functional gene names colored green and blue, respectively. Changes that have happened along the modern human lineage leading to functional and non-functional variants, for which a different functional/non-functional state exists in the other hominin species, are highlighted. The number of sequenced genomes are displayed. The three columns of OR genes along the modern human lineage represent the ancestral genes, Neanderthal gene losses and Denisovan gene losses.

A total of 10 functional modern human ORs from the HORDE reference database were found as pseudogenes in the high coverage Neanderthal genome (OR2L8, OR4X1, OR5AR1, OR5M11, OR5P3, OR6C74, OR7A5, OR8I2, OR10X1, OR13C4) (Table 1). By comparing these Neanderthal pseudogenes to known modern human OR loss of function SNPs [9], we found 7 of these ORs with identical non-sense mutations present both in Neanderthals and in a subset of modern humans (OR2L8, OR4X1, OR5AR1, OR5M11 OR6C74, OR8I2, OR10X1), while 3 have different Neanderthal specific loss of function mutations (OR5P3, OR7A5, OR13C4) (Figure 2). Three non-functional modern human ORs have functional Neanderthal homologs (OR1P1P, OR2J1P, OR9H1P). These Neanderthal mutations can be found as ‘rescued’ ORs in some modern humans [9].

The Denisovan genome shows evidence of 8 non-functional ORs with functional modern human homologs (OR2L8, OR2S2, OR2T8, OR4C12, OR5AC2, OR5M11, OR8I2, OR51Q1). These ORs cover 4 subfamilies, 1 from Class I and 3 from Class II. Identical non-sense mutations led to loss of function for 4 of these ORs (OR2L8, OR5M11, OR8I2, OR51Q1) in some modern humans [9], while 4 ORs (OR2S2, OR2T8, OR4C12, OR5AC2) have loss of function mutations unique to the Denisovan lineage (Table 1). Additionally, within the Denisovan genome, 5 ORs (OR1P1P, OR2J1P, OR5E1P, OR9H1P, OR13C1P) from Class II lack stop codons despite their presence within modern human OR orthologs. Three of these ORs (OR1P1P, OR2J1P, OR9H1P) have modern human polymorphisms that can ‘rescue’ these ORs.

The mutation leading to non-functionality for OR2L8, OR8I2 and OR5M11 are shared between the Denisovan and Neanderthal, with the same mutation existing in some modern humans. This may indicate that the ancestral state for these ORs in the Neanderthal-Denisovan-modern human most recent common ancestor is non-functional, with modern humans regaining function post-divergence. The mutation that retains function for OR1P1P, OR2J1P and OR9H1P is shared between Neanderthals, Denisovans and some modern humans. This may indicate an ancestral functional state, with a loss of function happening along the modern human lineage. However, without a larger population sampling of extinct genomes for each hominin species, it is difficult to discriminate among alternative evolutionary trajectories.

The deamination of cytosine-to-thymine and guanine-to-adenine can cause a large number of sequencing errors in studies using ancient DNA [18]. Three Neanderthal (OR5AR1, OR6C74, OR10X1) and 3 Denisovan apparent loss of function mutations (OR4C12, OR5AC2, OR51Q1) potentially could have resulted from such deamination (Table 1). While it may be difficult to differentiate between real loss of function and deamination-induced damage, 2 of these ORs (OR6C74, OR10X1) show the same loss in the 1× genome for Neanderthals, suggesting that it is a real mutation event as opposed to deamination. None of the potential deamination events in the 30× Denisovan genome have coverage in the 1× genome version [19], however one of these mutations can be found as a variant of its modern human homolog (OR51Q1).

**Figure 3:**
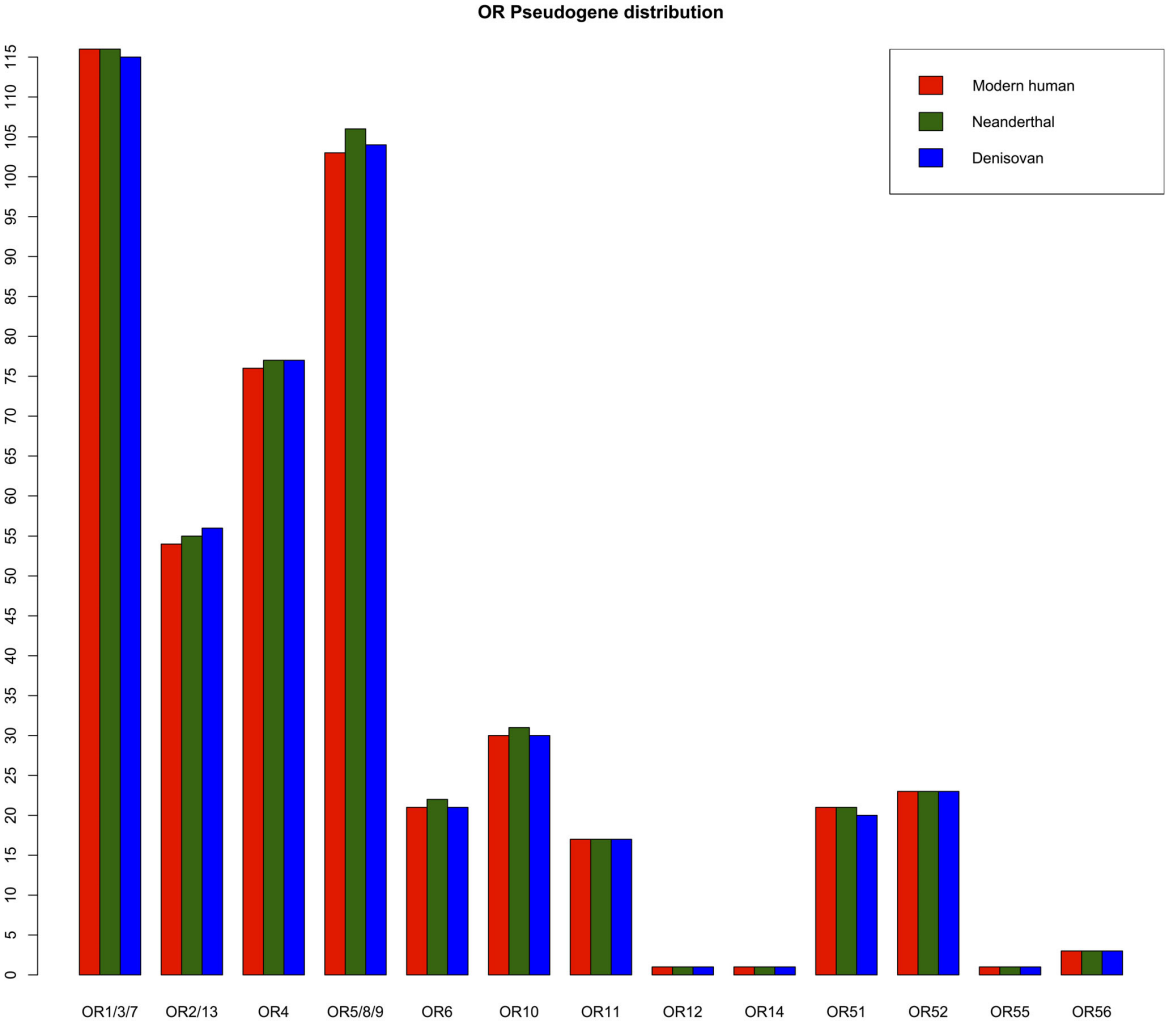
The distribution of OR pseudogenes for modern human (HORDE build #43), Neanderthal and Denisovan. The Denisovan OR families 2/13, 4, 5/8/9 and 51 show more non-functional ORs than in modern humans, while the Neanderthal has a higher number of non-functional ORs in families 2/13, 4, 5/8/9, 6 and 10 in comparison to modern humans.

There are 466 and 433 non-functional ORs in the modern human and chimpanzee genomes respectively. Of these, 99 modern human non-functional ORs have conserved stop codons also present in the chimpanzee, suggesting they were inherited as pseudogenes from the human-chimpanzee common ancestor and are therefore most likely present as inherited pseudogenes in the Neantherthal and Denisovan lineages. These data suggest at least 473 (466 modern human pseudogenes with an additional 10 pseudogenes, minus 3 functional Neanderthal ORs that are pseudogenes in modern humans) and 469 ORs are non-functional within the Neanderthal and Denisovan genomes respectively (Figure 3).

Based on DNA sequence analysis, the divergence time between Denisovans and Neanderthals is 640,000 years [5]. The divergence of Denisovans, Neanderthals and modern humans from their most recent common ancestor is estimated at 804,000 years ago [5]. The relatively recent divergence time between modern humans, Neanderthals and Denisovans suggests a rapid pseudogenization for these ORs. Periods of cold climatic conditions such as those of the middle-late Pleistocene [4] may have caused environmental pressures that could potentially play a role in loss of OR function. Considering that a minimum odorant detection threshold must be met to detect a smell in modern humans [3], in conjuncture with the effect of cold temperatures limiting odor volatility [20], it is possible that the loss of 10 Neanderthal and 8 Denisovan ORs may have had an affect on odorant perception for these hominin species. The loss of 10 ORs may be related to the decreased size of the olfactory bulb in Neanderthals, however inferring how gene loss may affect phenotype in extinct species remains challenging. Further research into how different odors bind to different receptors [21] may shed further light on this issue. This is an ongoing challenge in comparative sensory genomics.

This analysis of OR genes suggests that modern humans, Neanderthals and Denisovans were subject to a number of changes in their respective OR repertoires. As more ancient genomes are sequenced, such analyses may provide a better picture of how sensory perception has evolved, particularly in species for which little phenotypic information is known.
